# The High Interfacial Activity of Betaine Surfactants Triggered by Nonionic Surfactant: The Vacancy Size Matching Mechanism of Hydrophobic Groups

**DOI:** 10.3390/molecules30112413

**Published:** 2025-05-30

**Authors:** Guoqiao Li, Jinyi Zhao, Lu Han, Qingbo Wu, Qun Zhang, Bo Zhang, Rushan Yue, Feng Yan, Zhaohui Zhou, Wei Ding

**Affiliations:** 1 College of Chemistry and Chemical Engineering, Northeast Petroleum University, Daqing 163318, China; liguoqiao_2001@126.com; 2 No.2 Oil Production Plant, Daqing Oilfield Corp. Ltd., Daqing 163414, China; 18904893123@189.cn (J.Z.); wuqingbo07@petrochina.com.cn (Q.W.); 3 State Key Laboratory of Enhanced Oil & Gas Recovery (PetroChina Research Institute of Petroleum Exploration & Development), Beijing 100083, China; hanlu1991@petrochina.com.cn (L.H.); zhangqun1980@petrochina.com.cn (Q.Z.); zhangbo0804@petrochina.com.cn (B.Z.); 4 School of Chemistry, Tiangong University, Tianjin 300387, China; yue2021@outlook.com (R.Y.); yanfeng@tiangong.edu.cn (F.Y.)

**Keywords:** interfacial tension, betaine, nonionic surfactant, mixed adsorption, oil solubility, water solubility

## Abstract

Alkyl sulfobetaine shows a strong advantage in the compounding of surfactants due to the defects in the size matching of hydrophilic and hydrophobic groups. The interfacial tensions (IFTs) of alkyl sulfobetaine (ASB) and xylene-substituted alkyl sulfobetaine (XSB) with oil-soluble (Span80) and water-soluble (Tween80) nonionic surfactants on a series of n-alkanes were studied using a spinning drop tensiometer to investigate the mechanism of IFT between nonionic and betaine surfactants. The two betaine surfactants’ IFTs are considerably impacted differently by Span80 and Tween80. The results demonstrate that Span80, through mixed adsorption with ASB and XSB, can create a relatively compacted interfacial film at the n-alkanes–water interface. The equilibrium IFT can be reduced to ultra-low values of 5.7 × 10^−3^ mN/m at ideal concentrations by tuning the fit between the size of the nonionic surfactant and the size of the oil-side vacancies of the betaine surfactant. Nevertheless, Tween80 has minimal effect on the IFT of betaine surfactants, and the betaine surfactant has no vacancies on the aqueous side. The present study provides significant research implications for screening betaine surfactants and their potential application in enhanced oil recovery (EOR) processes.

## 1. Introduction

Crude oil is essential to both industrial chemical synthesis and economic growth [1]. After primary and secondary oil recovery, nearly 60% of the crude oil is still trapped in gaps and pores of rocks [2]. The development of advanced enhanced oil recovery (EOR) techniques has emerged as a critical research priority in oil fields. Extensive research over recent decades has established four principal EOR techniques: chemical flooding [3], gas flooding [4], microbial flooding [5], and thermal recovery [6]. Among them, chemical flooding is the most widely used EOR method [7]. Injecting appropriate surfactants can significantly reduce the oil/water interfacial tension (IFT), consequently reducing the capillary resistance. In order to effectively drive the residual crude oil from the reservoir, the IFT of the oil–water interface must reach an ultra-low level (IFT < 10^−2^ mN/m) [8].

In general, the greater the polarity differences between the two phases, the greater the IFT. Oil and water have opposite polarities, and therefore, there is a high IFT between them. Surfactants are a class of interfacial active substances with both hydrophilic and lipophilic groups in the molecular structure. Surfactants effectively interact with both the aqueous and oil phases. Their hydrophilic portions exert robust interactions with the aqueous phase, while their hydrophobic portions similarly display considerable interaction with the oil phase. These properties allow surfactants to serve as a comparable “bridge” that facilitates a seamless transition between the molecular interactions of the oil and aqueous phases.

Surfactants used in EOR include anionic surfactants, nonionic surfactants, and zwitterionic surfactants. Among them, anionic surfactants are extensively studied in EOR because of their high interfacial activity, good temperature resistance, low adsorption in reservoirs, and cheap production cost [9]. Nevertheless, owing to the presence of anions in their hydrophilic group, anionic surfactants are more likely to combine with divalent cations like Ca^2+^ and Mg^2+^ to form precipitates, limiting their application in high calcium and magnesium reservoirs [10]. The application of nonionic surfactants in tertiary oil recovery has been extensively studied due to their excellent emulsification, permeability, and stability, as well as their resistance to the influence of Ca^2+^ and Mg^2+^ in hard water [11]. However, nonionic surfactants are not suitable for high-temperature reservoirs, as their solubility drastically decreases when the temperature rises above their cloud point [12]. Zwitterionic surfactants have received considerable attention in tertiary oil recovery because of their excellent salt and temperature resistance, broad pH adaptation, excellent emulsifying and dispersing capabilities, and high compatibility with other surfactants [13].

Betaine is a typical zwitterionic surfactant, and its oil displacement characteristics have been extensively investigated [14]. Lv et al. [15] systematically evaluated the interfacial properties of three alkyl betaine surfactants (LSB, MSB, and BSB) with different hydrophobicity, and investigated their oil displacement efficiency through microscopic visualization of oil displacement experiments. The results show that all three surfactants can effectively reduce the IFT, with ultra-low IFT (<10^−2^ mN/m) attained when the appropriate branch chain matches with the active components in crude oil. In the homogeneous model, betaine surfactants can form a strong interfacial film to play an in situ plugging role, which improves the recovery rate and sweep efficiency. Zhou et al. [16] investigated the IFT of two distinct hydrophobic betaines (ASB and BSB) and a model oil, discovering that the hydrophilic groups (anion–cation part and hydroxyl group) of alkyl betaine were adsorbed at the interface between oil and water in a lying manner. This leads to a greater occupation area of the hydrophilic group compared to the hydrophobic group, making it difficult for alkyl betaine surfactants to form a relatively tight adsorption film at the interface layer, and the IFT against n-alkanes such as decane is unlikely to reach ultra-low values [17]. The inherent size disparity between the hydrophilic group and the lipophilic group in betaine molecules creates unique opportunities for achieving ultra-low IFT through strategic combination with complementary surface-active components, enabling precise regulation of interfacial film structure.

Linear alkyl betaine is a widely used and cost-effective zwitterionic surfactant. However, the size difference between the hydrophobic alkyl chain and the hydrophilic group is large, causing it to only have a synergistic effect with the large-sized active components in the crude oil. This problem can be solved by surfactant compounding. In a study of the mechanism of electrolyte on the IFT of alkyl sulfobetaine (ASB) and petroleum sulfonate (PS) mixed solution, Sun et al. [18] discovered that a synergistic interaction of ASB and PS due to electrostatic attraction and the electrolyte could affect the synergistic interaction between ASB and PS by affecting the size of the PS molecules. Zhong et al. [19] studied the interaction between different ionic surfactants and alkyl sulfobetaine (ASB) and found that ASB had an antagonism with cationic surfactants and a synergism with anionic surfactants, both of which were attributed to electrostatic interaction. The synergistic effect with anionic surfactants is affected by the matching of alkyl chain length and the size compatibility of hydrophobic groups. Zhang et al. [20] compounded carboxyl betaine (CnZC) with extended surfactant (C_16_P_3_E_6_S) and found that there was a synergistic effect between them, which could reduce the IFT to an ultra-low level, and the recovery rate was increased by 20.4%. Pei et al. [21] discovered that nonylphenol ethoxylate carboxylate (NPEC) and oleic amide propyl betaine (OAPB) exhibited a synergistic effect on reducing IFT and enhancing emulsifying ability. This synergistic effect arises from the electrostatic interaction between them, which contributes to forming a denser adsorption film. Zhong et al. [22] proposed the necessary conditions for achieving ultra-low IFT in their study of the IFTs of alkyl carboxyl betaine (ACB) mixed with various anionic–nonionic surfactants; a suitable hydrophilic–lipophilic balance is the basis, and the size compatibility on both sides of the oil and water is the key factor. These reports primarily focus on the research of mixtures of alkyl betaine surfactants with anionic and anionic–nonionic surfactants.

Nonionic surfactants, due to their controllable hydrophilic–lipophilic balance and high salt tolerance, play a pivotal role in the phase inversion process of emulsions [23]. These properties make them particularly valuable in composite oil displacement systems. For instance, the impact of nonionic aliphatic alcohol ethoxylates (C_12_E_3_) on the interfacial activity of anionic–nonionic fatty alcohol polyoxyethylene carboxylate (C_12_EO_3_C) was examined by Liu et al. [24]. They found that under Na+ conditions, ultra-low IFT was obtained by C_12_E_3_ and C_12_EO_3_C mixed adsorption on specific alkanes. In the presence of Ca^2+^, the mechanism of reducing IFT changed from mixed adsorption to the regulation of hydrophilic–lipophilic balance with the increase of C_12_E_3_ concentration in the mixed system. Recently, Zhang et al. [25] explored the synergistic effect of nonionic alkyl polyglycoside (APG0810) and extended betaine surfactant (C_12_P_3_E_3_NS), and found that under the salinity of 10wt%, C_12_P_3_E_3_NS and APG0810 with a molar ratio of 4:3 had excellent crude oil emulsification and wettability modification ability. Nevertheless, there are few reports on the interaction between nonionic surfactants and betaine zwitterionic surfactants, and the related mechanism is still unclear.

In order to overcome the problem of low interfacial activity caused by the size mismatch of hydrophilic/hydrophobic groups of betaine surfactants, in this study, straight-chain alkyl sulfobetaine (ASB) and xylene-substituted branched alkyl sulfobetaine (XSB) were studied. They are unlikely to achieve ultra-low IFT when they exist alone, but they have high compatibility with other surfactants. Therefore, nonionic surfactants with different structures were introduced to regulate the structure of ASB and XSB at the interface between oil and water. The effects of oil-soluble and water-soluble nonionic surfactants on the oil–water IFT of ASB and XSB were investigated, and the different interaction mechanisms of oil-soluble and water-soluble nonionic surfactants on betaine surfactants were explained.

## 2. Results and Discussion

### 2.1. Effect of Surfactant Concentration on IFT

**Figure 1:**
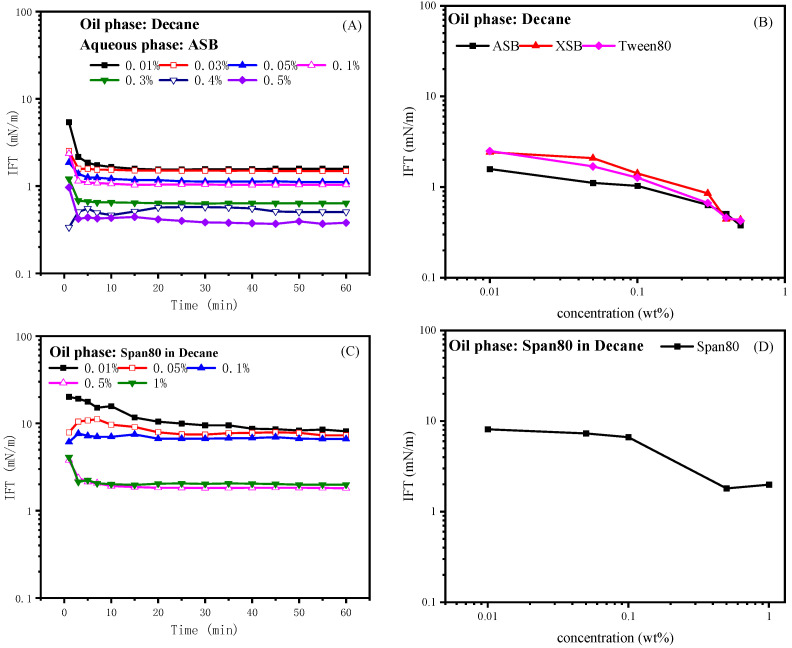
Dynamic IFTs of the ASB solution (**A**), equilibrium IFTs of the ASB, XSB, and Tween80 solution (**B**), dynamic IFTs of *n*-decane with Span80 against simulated water (**C**), and equilibrium IFTs of *n*-decane with Span80 against simulated water (**D**).

Surfactant adsorption at the interface dramatically reduces the interfacial energy, resulting in a lower IFT [26,27,28]. Although their molecular structure is different, ASB, XSB, Span80, and Tween80 are surfactants with hydrophilic groups and hydrophobic groups. They are adsorbed at the interface between oil and water and reduce the IFT. Initially, the impact of the four surfactants on oil–water IFT when used in isolation was assessed, as depicted in Figure 1. Figure 1A,C presents the dynamic IFT curves of ASB and Span80 at the interface of decane–water. Figure 1B,D shows the equilibrium IFT curves of ASB, XSB, Span80, and Tween80.

As observed in Figure 1A, the dynamic IFT curve of ASB is of the most common type, an “L” type curve [29]. Due to the presence of a concentration gradient as the oil–water phases begin to contact, the surfactant quickly diffuses from the water bulk to the oil–water interface and adsorbs on the interface, resulting in the rapid decrease in IFT. An increase in surfactant concentration at the interface accelerates the diffusion rate of surfactants from the interface to the bulk phase. Simultaneously, the rate at which IFT decreases is retarded. Eventually, a dynamic equilibrium between adsorption and desorption is established, leading to the stabilization of the IFT. As can be observed in Figure 1B, the equilibrium IFT values of ASB are all greater than 0.1 mN/m, indicating that ASB has low activity at the interface between decane and water. In general, the ability of surfactants to reduce IFT depends on two factors: the concentration of surfactant on the oil–water interface and the differences between the sizes of hydrophilic groups and hydrophobic groups (size matching) [30]. Only when the concentration of surfactant on the oil–water interface is relatively high and the size of hydrophilic and lipophilic groups in the molecular structure match can surfactants fully replace oil and water molecules at the interface, allowing for the realization of ultra-low IFT [31]. For the ASB studied in this study, it is difficult to form a dense adsorption film because of the mismatch in size between hydrophilic and hydrophobic groups. Therefore, ASB cannot achieve an ultra-low IFT.

Similarly, XSB also has the problem of size mismatch between hydrophilic and hydrophobic groups, and it is also unlikely to form a dense adsorption film. Therefore, as shown in Figure 1B, it is not difficult to understand that the IFT of XSB is greater than 0.1 mN/m in the experimental range.

Tween80, as a conventional water-soluble surfactant, does not exhibit very high interfacial activity. As shown in Figure 1B, within the studied concentration range, the IFT values are all above 0.1 mN/m.

The HLB value of Span80 is 4.3, and its solubility in water is very small; therefore, it can only form a dispersion system in water. Span80 was dissolved in the oil phase, and its IFT with water was measured. The results are shown in Figure 1C,D. It can be observed that the equilibrium IFT value is above 1 mN/m, indicating that Span80 has low surface activity when present alone.

### 2.2. Effect of Oil-Soluble Nonionic Surfactants on the IFT of Betaine

#### 2.2.1. Effect of Span80 on the IFT of Betaine ASB

To address the issue of mismatched sizes between the hydrophilic and lipophilic groups of betaine surfactants, we investigated the effect of Span80 on the interfacial activity of ASB at the interface between oil and water in this section.

**Figure 2:**
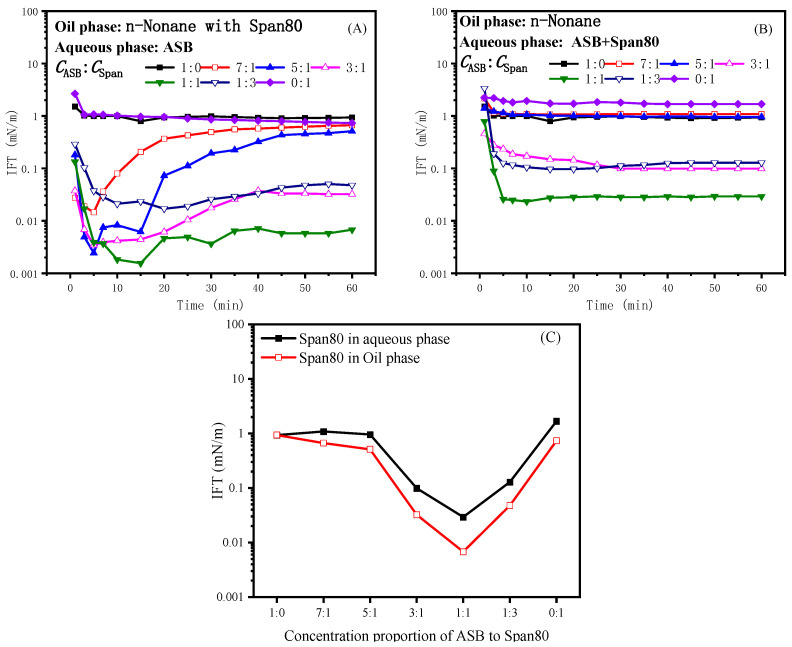
Dynamic IFTs of ASB solutions against n-nonane with Span80 (**A**), dynamic IFTs of mixed solutions of ASB and Span80 (**B**), and equilibrium IFTs of the mixed system (**C**). The total concentration of the mixed system solution is 0.4wt%.

In the absence of the solubilizing effect of other surfactants, Span80 has a very low solubility in water. However, in a practical oil displacement system, surfactants are dissolved in water. Consequently, an initial investigation was conducted to examine the disparity in the impact of Span80 on the IFT of ASB at the oil–water interface, comparing its effects in the oil and water phases, and the result is shown in Figure 2.

Figure 2A illustrates the dynamic IFTs when Span80 and ASB are dissolved separately in the oil phase and the water phase, respectively, while Figure 2B exhibits the dynamic IFTs when both Span80 and ASB are dissolved in the aqueous phase. In Figure 2A, a distinct “V” shape curve can be observed when the ASB:Span80 ratio is 7:1 and 5:1. Herein, a small amount of Span80 contained in the oil phase can be regarded as an interfacial active substance. At the beginning, ASB molecules adsorb at the interface, with their hydrophilic groups gradually transitioning from an upright position to a flat position. Meanwhile, Span80 diffuses from the oil phase to the interface and inserts itself into the available spaces on the oil side of the ASB molecules, forming a dense composite interfacial adsorption film, which significantly reduces the IFT value. As the interface ages, the hydrophilic groups of ASB molecules become completely flat, and the dense interfacial film becomes loose, leading to an increase in the IFT value.

From Figure 2C, it is evident that the effect of Span80 dissolved in the oil phase and water phase on the IFT of ASB is similar, but the IFT value is greater when Span80 is dispersed within the water phase. This is because when Span80 is dissolved in the oil phase, Span80 diffuses directly to the interface, where it combines with ASB to create a mixed adsorption film. However, when Span80 and ASB are co-dissolved in the aqueous phase, Span80 molecules need to be distributed to the oil phase through the interface layer before forming mixed adsorption with ASB from the oil side. This complex adsorption process undoubtedly hinders the formation of a dense interface layer of Span80 and ASB, resulting in an increase in IFT. Nevertheless, with the change of the ASB:Span80 ratio, the trend of IFT in the two cases was completely consistent, indicating that Span80 dissolved in the water phase and oil phase had little effect on the experimental results. Considering that surfactants are dissolved in water in actual oilfield production applications, Span80 and ASB were mixed and dissolved in water for subsequent experiments.

To our surprise, it can be seen in Figure 2C that when the ASB:Span80 ratio is 3:1, 1:1, and 1:3, the IFT value is significantly reduced. This result indicated that Span80 and ASB had an obvious synergistic effect under the abovementioned ratio conditions, which may be the result of mixed adsorption of Span80 and ASB. Generally, for a binary surfactant system, there are two main ways to affect IFT [24]. One is the mixed adsorption: two surfactants with different structural characteristics can be mixed and adsorbed on the oil-water interface, forming a more compact adsorption film on the interface, thereby changing the IFT. The other is a suitable hydrophilic–lipophilic balance (HLB). That is, the change of the ratio of the two surfactants can regulate the HLB value of the oil–water system. When the distribution of the two surfactant molecules in the water phase, oil phase, and interface is appropriate, the IFT value can be decreased significantly. For the latter, the IFTs between alkanes with various alkane carbon numbers (ACN) and surfactant solutions can be measured. The ACN with the lowest IFT is referred to as n_min_, which can be used to measure the HLB value [32]. The smaller the n_min_ value, the higher the hydrophilicity. The change of HLB of the mixed-surfactant solution can be determined by measuring the n_min_ value.

**Figure 3:**
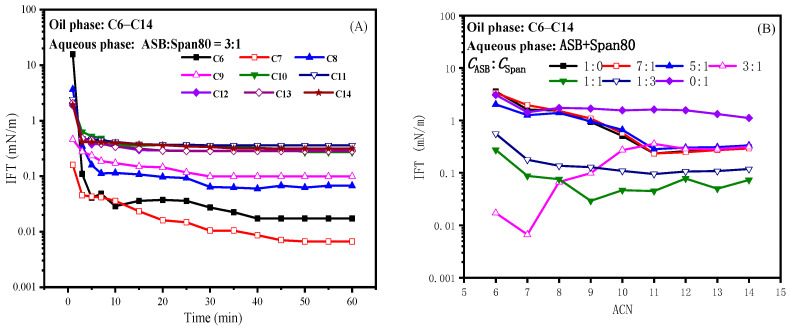
Dynamic IFTs (**A**) and equilibrium IFTs (**B**) of the mixed solution of ASB and Span80. The total concentration of the mixed system solution is 0.4wt%.

In order to explore whether the abovementioned phenomenon of Span80 reducing the IFT of ASB is dominated by hydrophilic–lipophilic balance or mixed adsorption, the IFT of the ASB and Span80 mixed solution with n-alkanes of different ACN was tested. For simplicity, the dynamic IFT is given in a mixed system ratio of ASB:Span80 = 3:1 (as shown in Figure 3A), and the equilibrium IFT is plotted as a function of ACN in Figure 3B. The change trend of dynamic IFT in Figure 3A is consistent with the dynamic IFT described in Section 2.1, and the principle is not repeated here. As evident from Figure 3B, the interfacial activity of both ASB and Span80 is not high when present individually, and the IFTs are higher than 0.1 mN/m. However, in the binary mixture system of ASB and Span80, the IFTs can be reduced at the ratio of ASB:Span80 = 3:1, 1:1, and 3:1. At lower concentrations of Span80 (such as the ratio of ASB:Span80 = 7:1 and 5:1), Span80 exerts essentially no effect on the IFT of ASB. With the increase of Span80 concentration, a strong synergistic effect is observed. At the ratio of ASB:Span80 = 3:1, the IFT of the system with n-heptane reaches 6.7 × 10^−3^ mN/m. Furthermore, the overall ACN curve of the mixed system gradually shifts upwards, indicating a diminution in the synergistic effect with the further increase of the proportion of Span80 (the ratio of ASB:Span80 = 1:1 and 1:3). Additionally, one can observe from Figure 3B that the *n*_min_ of ASB is 11, and the *n*_min_ of Span80 is greater than 14, both of which have strong oil solubility. If controlled by the hydrophilic–lipophilic balance, the *n*_min_ of the mixed solution should fall between the *n*_min_ values of ASB and Span80. However, the experimental results do not conform to this phenomenon. In the most significant synergistic effect of the 3:1 ratio of the mixed system, the value of *n*_min_ is 7. In other proportions with synergistic effects, *n*_min_ is also less than 11. This implies that the cooperative action between ASB and Span80 is governed by mixed adsorption, where the *n*_min_ does not represent the hydrophilic–lipophilic balance. The sizes of the hydrophilic group and the hydrophobic group of the betaine ASB molecules adsorbed on the interface are quite different, forming “space vacancies” on the oil side of the interface. Span80 tends to be distributed to the oil phase due to its strong oil solubility. Based on the aforementioned points, the adsorption mechanism of ASB and Span80 may be as follows: Span80 adsorbs with ASB from the oil phase side through the space vacancy on the oil phase side between ASB molecules, forming a more compact adsorption film and decreasing the IFT.

#### 2.2.2. Effect of Span80 on the IFT of Betaine XSB

Betaine ASB is a linear alkyl sulfobetaine, while XSB is a branched alkyl sulfobetaine. Obviously, the size difference between the hydrophilic and hydrophobic groups of XSB is smaller than that of ASB. Consequently, the space vacancy on the oil side of XSB is more confined compared to ASB [33]. By studying the IFT of the mixed system of Span80 and XSB with n-alkanes of different ACN, we can further verify whether the space vacancy on the oil phase side has an effect on the synergistic effect between oil-soluble nonionic surfactants and betaine surfactants.

**Figure 4:**
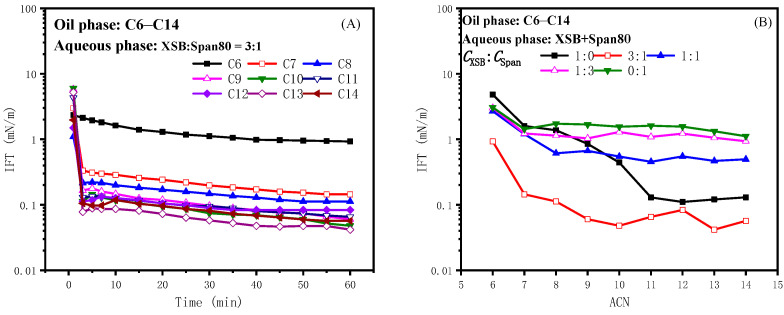
Dynamic IFTs (**A**) and equilibrium IFTs (**B**) of the mixed solution of XSB and Span80. The total concentration of the mixed system solution is 0.4wt%.

The dynamic IFTs and equilibrium IFTs of the mixed system of Span80 and XSB are shown in Figure 4. Figure 4A demonstrates dynamic IFT, which is consistent with the previous results. In Figure 4B, within the binary system of Span80 and XSB, the IFT of the mixed system reaches its minimum value of 4.2 × 10^−2^ mN/m at the ratio of XSB:Span80 = 3:1, indicating a certain synergistic effect. However, with an increase in Span80’s concentration in the combined system, the IFT value of the mixture also climbs. At the ratio of XSB:Span80 = 1:1 and 1:3, the ACN curve of the mixed system is between the ACN curves of XSB and Span80, suggesting that the synergistic effect of Span80 and XSB disappeared.

Comparing Figure 4B with Figure 3B, it is evident that the synergistic effect between Span80 and XSB is not as strong as that between Span80 and ASB, and the proportionate range of the synergistic interaction is narrower. This is attributed to the fact that the hydrophobic group of ASB possesses a straight chain, whereas the lipophilic group of XSB features a dimethylphenyl side chain. Regarding adsorption at the interface between oil and water, the space vacancy of XSB on the oil side is smaller than that of ASB. The molecular size of Span80 is better matched with the space vacancy size of ASB on the oil side, but it is larger than the space vacancy size of XSB. Consequently, Span80 cannot form a tight mixed adsorption film through the space vacancy of XSB on the oil side, resulting in an increase in IFT. The abovementioned results indicate that when the spatial vacancy of betaine molecules on the oil side is poorly matched with the oil-soluble nonionic size, the synergistic effect is weakened.

#### 2.2.3. Effect of Propylene Glycol Monostearate on the IFT of Betaine XSB

**Figure 5:**
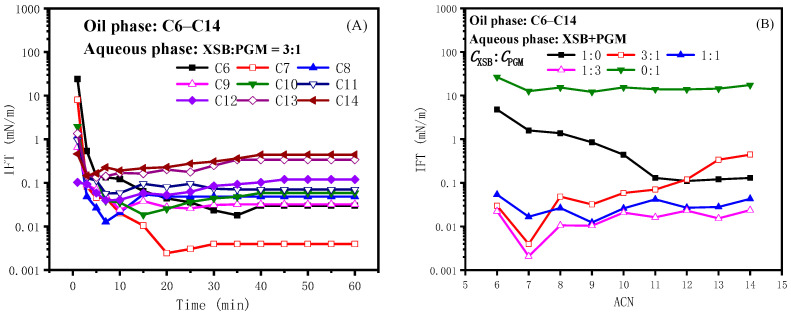
Dynamic IFTs (**A**) and equilibrium IFTs (**B**) of the mixed solution of XSB and PGM. The total concentration of the mixed system solution is 0.4wt%.

To further verify the matching characteristics between the size of oil-soluble nonionic surfactant and the size of space vacancy on the oil side of the betaine surfactant, PGM with a smaller hydrophilic head size was selected to be compounded with XSB, and the IFT of the mixed system with n-alkanes of different ACN was tested. The result is presented in Figure 5. Figure 5A shows the dynamic IFT curves of the mixed solutions of XSB and PGM, and Figure 5B presents the equilibrium IFT of the mixed solution. The IFT of PGM is higher than 10 mN/m, indicating that its interfacial activity is not high. However, when PGM is compounded with XSB, the IFT significantly decreases, and in a wide concentration and ACN range, the IFT is much lower than that of XSB. In particular, at the mass concentration ratio of XSB:PGM = 1:3, the IFT reaches 2.1 × 10^−3^ mN/m, indicating a significant synergistic effect between PGM and XSB.

#### 2.2.4. Synergistic Mechanism of Oil-Soluble Surfactants with Betaine ASB and XSB

The hydrophobic groups of PGM and Span80 are stearic acid and oleic acid, respectively, while the hydrophilic groups are propylene glycol and dehydrated sorbitol. Obviously, the hydrophobic groups of the two have the same alkyl carbon number, but the hydrophilic group of PGM is significantly smaller than that of Span80. PGM, with a smaller molecular size, can form a mixed adsorption film with XSB through the smaller space vacancies on the oil side of XSB, resulting in a strong synergistic effect at the interface between oil and water. This fully verifies the importance of matching the size of nonionic surfactants with the space vacancy on the oil side of alkyl betaine when combining oil-soluble nonionic surfactants with alkyl betaine surfactants.

**Figure 6:**
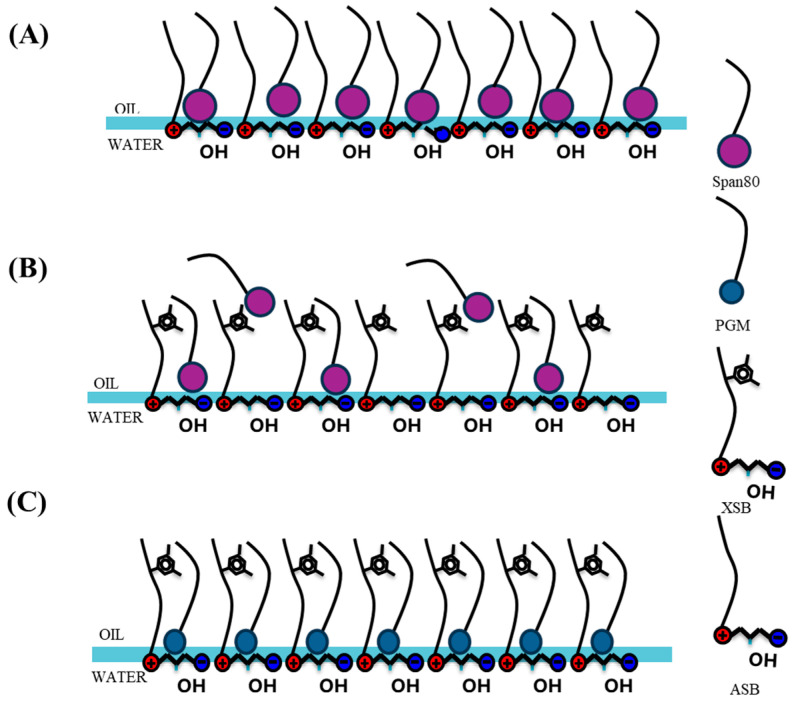
Schematic diagram of synergistic effect mechanism of oil-soluble nonionic surfactant and betaine. Span80 and ASB (**A**), Span80 and XSB (**B**), PGM and XSB (**C**).

Based on the aforementioned experimental findings, the mechanism diagram of the impact of oil-soluble nonionic surfactant on the IFT of betaine surfactant can be proposed. At an optimal concentration, Span80 molecules engage in a dense mixed adsorption with ASB by filling the space vacancies on the oil side of ASB, as shown in Figure 6A. At elevated concentrations of Span80, Span80 is excessively adsorbed through the space vacancy on the oil side of ASB, leading to a reduction in the compactness of the adsorption film and the weakening of the synergistic impact. For XSB, due to the smaller space vacancy, the size of Span80 is not consistent with the space vacancy size. The synergistic effect is weakened, and the effective range of the synergistic effect is narrowed (as shown in Figure 6B). Because PGM has a smaller hydrophilic group, the molecular size of PGM is more compatible with the space vacancy of XSB. PGM can adsorb with XSB at the interface through the space vacancy on the oil side, forming a tight adsorption film (as depicted in Figure 6C).

### 2.3. Effect of Water-Soluble Nonionic Surfactant on IFT of Betaine

From the analysis in Section 2.1 and Section 2.2, it can be seen that the hydrophilic groups of betaine surfactant molecules have both cationic and anionic groups, which are electrically neutral. The repulsive force of the hydrophilic regions is relatively small, and they are arranged closely in a flat manner at the interface (on the aqueous side). Therefore, the occupied area of hydrophilic and hydrophobic groups on both sides of the oil–water interface is quite different. It has been confirmed in Section 2.2 that the oil-soluble surfactants Span80 and PGM have a good matching effect with the space vacancies of ASB and XSB, respectively, which can lower the IFT to an ultra-low value. Therefore, can Tween80, a water-soluble surfactant with the same hydrophobic group but different hydrophilic groups as Span80, play a similar role in regulating the betaine adsorption film? In this part, the impact of water-soluble nonionic surfactant on the interfacial activity of the two betaine surfactants will be studied.

#### 2.3.1. Effect of Tween80 on IFT of Betaine ASB

**Figure 7:**
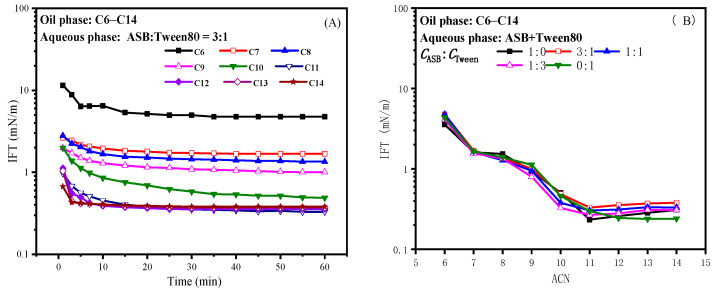
Dynamic IFTs (**A**) and equilibrium IFTs (**B**) of the mixed solution of ASB and Tween80. The total concentration of the mixed system solution is 0.4wt%.

Figure 7 illustrates the dynamic and equilibrium IFT of the mixed system of Tween80 and ASB. On the one hand, under a fixed concentration, the IFT gradually decreases as the ACN increases. However, once the ACN reaches 11, the IFT almost remains unchanged with increasing ACN. On the other hand, under a fixed ACN value, the IFT of the binary systems with different ratios is almost equal, indicating that the interaction between ASB and Tween80 does not exhibit any synergistic or antagonistic properties within the experimental range. The HLB value of Tween80 is 15.0, which has strong hydrophilicity and is mainly distributed in the aqueous phase, while ASB can form a tight adsorption on the side of the aqueous phase, which hinders the diffusion of Tween80 to the interface layer. Therefore, Tween80 has no effect on the IFT of the ASB solution.

**Figure 8:**
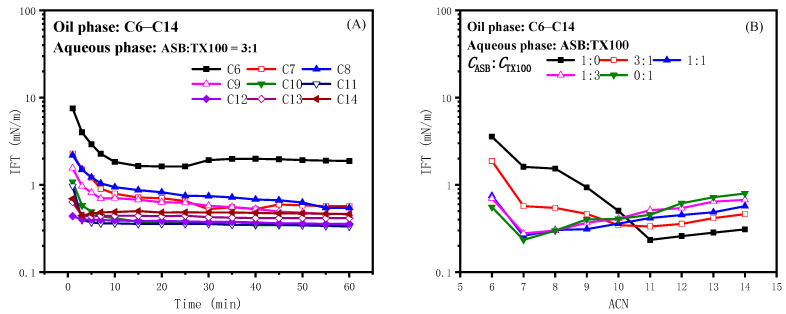
Dynamic IFTs (**A**) and equilibrium IFTs (**B**) of the mixed solution of ASB and TX100. The total concentration of the mixed system solution is 0.4wt%.

In order to further verify the abovementioned conjecture, TX100, with smaller hydrophilic groups, was selected to explore the effect of water-soluble nonionic surfactants on ASB by the same research method. The outcomes are presented in Figure 8. As can be observed, when the ACN value is less than 10, the IFT of the mixed system of TX100 and ASB is close to that of TX100 alone, while when the ACN value is greater than 10, the IFT of the mixed system is consistent with that of ASB alone. With the increase of TX100 content in the mixed system, the ACN value of the mixed system of ASB and TX100 shifted to the low alkane carbon number, which proved that it was the effect of enhancing water solubility rather than mixed adsorption. In addition, the IFT of the ASB and TX100 mixed system is basically between the IFT of ASB and TX100 alone, indicating that there is no obvious synergistic effect between ASB and TX100. The above results suggest that water-soluble nonionic compounds with smaller hydrophilic groups also cannot form mixed adsorption with ASB through the aqueous phase side.

Based on the above discussion, the possible interface arrangement of Tween80 and ASB was proposed. That is, Tween80 has strong water solubility and is more distributed in the water phase, while ASB does not have space vacancies on the water phase side, resulting in Tween80 molecules being unable to form mixed adsorption with ASB from the water phase side. Therefore, Tween80 has little effect on the IFT of ASB.

#### 2.3.2. Effect of Tween80 on the IFT of Betaine XSB

**Figure 9:**
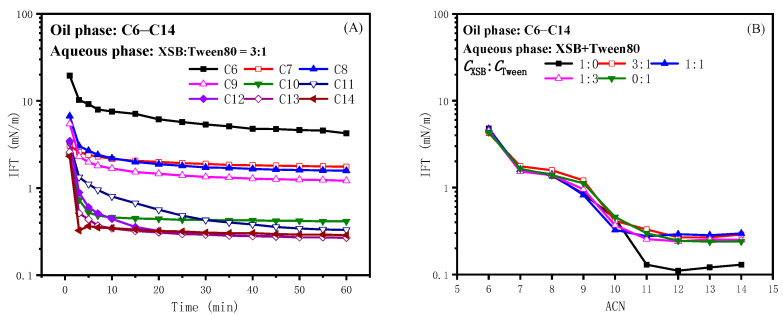
Dynamic IFTs (**A**) and equilibrium IFTs (**B**) of the mixed solutions of XSB and Tween80. The total concentration of the mixed system solution is 0.4wt%.

In order to further verify the interaction of water-soluble nonionic Tween80 with betaine, the IFT between the mixed system of Tween80 and XSB with water was measured. The findings are depicted in Figure 9. It can be observed that Tween80 exerts only a minimal impact on the IFT of XSB. Only at high ACN value, the IFT of the mixed system is slightly higher than that of Tween 80 and XSB alone. This is because XSB has strong oil solubility, and its ability to distribute to the oil phase is stronger than that of ASB. The XSB molecule at the interface is situated in closer proximity to the oil phase. Therefore, in the presence of high alkane carbon number oil phase, Tween80 can show a certain competitive adsorption capacity with XSB on the water phase side, and the IFT increases slightly. These results further confirmed that the water-soluble nonionic surfactant could not have a synergistic effect with the betaine through the side of the aqueous phase due to the tight adsorption of betaine surfactant on the water side.

## 3. Materials and Methods

### 3.1. Materials

**Scheme 1:**
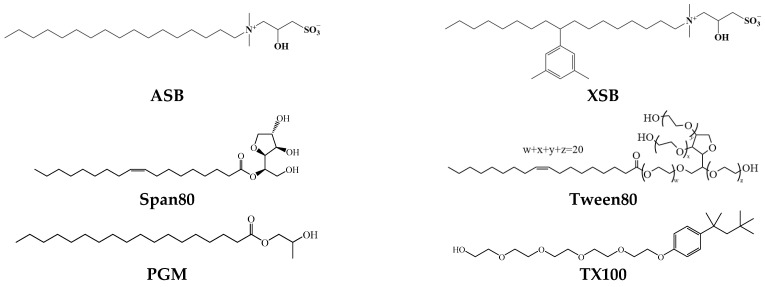
Structures of ASB, XSB, Span80, Tween80, PGM, and TX100.

Alkyl sulfobetaine (ASB) and xylene-substituted alkyl sulfobetaine (XSB) were synthesized by our laboratory with a purity of more than 95%. Span80, Tween80, Triton X-100 (TX100), and propylene glycol monostearate (PGM) were purchased from Aladdin (Beijing, China), with a purity of more than 98%. The structural formulas of the abovementioned surfactants are shown in Scheme 1. The oil phase used is C6-C14 n-alkanes, which are all analytically pure and are also purchased from Aladdin Scientific (Beijing, China).

The surfactant solutions used in the experiment were all prepared with simulated water, and the chemicals used to prepare simulated water, such as CaCl_2_, MgCl_2_·6H_2_O, and NaCl, were all analytically pure. Table 1 displays the composition of simulated water.

### 3.2. Apparatus and Method

The IFT data presented in this study were measured using the spinning drop method (CNGTX interfacial tension meter, Beijing Shengweiji Technology Co., Ltd., Beijing China) as described previously [16,34]. The rotational speed was 5000 r/min, and the experimental temperature was 75 °C. Before the experiment, the amplification factor was measured by the standard wire, and pure water and air were used as the control samples for calibration. During the test, when the IFT remained unchanged within 30 min, it was considered that the IFT had reached equilibrium. The parallel error between the three experiments is less than 5%. When the IFT value is less than 1 mN/m, the measurement error of the IFT value is less than ±5%.

## 4. Conclusions

Herein, the effects of oil-soluble and water-soluble nonionic surfactants on the IFT of two betaine surfactants, ASB and XSB, have been studied. The IFTs of the mixed systems of nonionic surfactants and betaine surfactants with different structures against n-alkanes were investigated. Based on the abovementioned experimental results and theoretical assessments, the following conclusions can be obtained:

(1) Due to the betaine molecules being flat at the oil/water interface, where the hydrophilic groups occupy a larger space than the hydrophobic groups, achieving ultra-low IFT values with pure betaine surfactants remains challenging. The oil-soluble nonionic surfactant Span80 can occupy the vacancy in the hydrophobic part of ASB and XSB and reduce the equilibrium IFTs. Ultra-low IFTs can be achieved in the ASB/Span80 mixed system by optimizing the ratio of ASB to Span80.

(2) Tween80, as a water-soluble nonionic surfactant, has an HLB value of 15.0 and a high distribution ratio in the aqueous phase. However, the hydrophilic groups of ASB and XSB are closely arranged on the water phase side of the interface, and there is no vacancy. Tween80 is unlikely to form mixed adsorption from the aqueous side through a relatively tight film of betaine, and therefore, it has little effect on the IFT of ASB and XSB.

(3) The synergistic effect between oil-soluble nonionic surfactants and betaine can be influenced by adjusting the degree of matching between the size of the oil-soluble surfactants and the spatial vacancy on the betaine’s oil-phase interface. The space vacancy of XSB on the oil side is smaller than that of ASB. The larger size of Span80 results in a poor match with the vacancy of XSB, thereby diminishing the synergistic effect and increasing the IFT. The smaller size of PGM can match the vacancies of XSB to form a more compact adsorption film, and the IFTs reached ultra-low values once more.

(4) The synergistic effect of oil-soluble nonionic surfactant and betaine, affected by the size compatibility, can become the guiding principle for the future design of betaine-related oil displacement formulation.

## Figures and Tables

**Table 1:** Ion composition of simulated water.

Cl^−^	Na^+^	Ca^2+^	Mg^2+^	TDS
11,699	6921	413	102	19,135

## Data Availability

No new data were created or analyzed in this study. Data sharing is not applicable to this article.
